# Graphene Quantum Dots Electrochemistry and Sensitive Electrocatalytic Glucose Sensor Development

**DOI:** 10.3390/nano7100301

**Published:** 2017-09-29

**Authors:** Sanju Gupta, Tyler Smith, Alexander Banaszak, John Boeckl

**Affiliations:** 1 Department of Physics and Astronomy and Biotechnology Center, Western Kentucky University, 1906 College Heights Blvd, Bowling Green, KY 42101, USA; sgup@rocketmail.com; 2 The Gatton Academy of Mathematics and Science, 1906 College Heights Blvd, Bowling Green, KY 42101, USA; sgup77@gmail.com; 3 Air Force Research Laboratory, Wright-Patterson Air Force Base, Wright-PATT, OH 45433, USA; john.boeckl@us.af.mil

**Keywords:** graphene quantum dots, hydrothermal, electrochemistry, glucose oxidase, biosensing

## Abstract

Graphene quantum dots (GQDs), derived from functionalized graphene precursors are graphene sheets a few nanometers in the lateral dimension having a several-layer thickness. They are zero-dimensional materials with quantum confinement and edge site effects. Intense research interest in GQDs is attributed to their unique physicochemical phenomena arising from the sp^2^-bonded carbon nanocore surrounded with edged plane functional moieties. In this work, GQDs are synthesized by both solvothermal and hydrothermal techniques, with the optimal size of 5 nm determined using high-resolution transmission electron microscopy, with additional UV-Vis absorption and fluorescence spectroscopy, revealing electronic band signatures in the blue-violet region. Their potential in fundamental (direct electron transfer) and applied (enzyme-based glucose biosensor) electrochemistry has been practically realized. Glucose oxidase (GO*_x_*) was immobilized on glassy carbon (GC) electrodes modified with GQDs and functionalized graphene (graphene oxide and reduced form). The cyclic voltammetry, differential pulse voltammetry, and electrochemical impedance spectroscopy are used for characterizing the direct electron transfer kinetics and electrocatalytical biosensing. The well-defined quasi-reversible redox peaks were observed under various electrochemical environment and conditions (pH, concentration, scan rate) to determine the diffusion coefficient (D) and first-order electron transfer rate (*k*_ET_). The cyclic voltammetry curves showed homogeneous ion transport behavior for GQD and other graphene-based samples with D ranging between 8.45 × 10^−9^ m^2^ s^−1^ and 3 × 10^−8^ m^2^ s^−1^ following the order of GO < rGO < GQD < GQD (with FcMeOH as redox probe) < GO*_x_*/rGO < GO*_x_*/GO < HRP/GQDs < GO*_x_*/GQDs. The developed GO*_x_*-GQDs biosensor responds efficiently and linearly to the presence of glucose over concentrations ranging between 10 μM and 3 mM with a limit of detection of 1.35 μM and sensitivity of 0.00769 μA μM^−1^·cm^−2^ as compared with rGO (0.025 μA μM^−1^ cm^−2^, 4.16 μM) and GO (0.064 μA μM^−1^ cm^−2^, 4.82 μM) nanosheets. The relatively high performance and stability of GQDs is attributed to a sufficiently large surface-to-volume ratio, excellent biocompatibility, abundant hydrophilic edges, and a partially hydrophobic plane that favors GO*_x_* adsorption on the electrode surface and versatile architectures to ensure rapid charge transfer and electron/ion conduction (<10 ms). We also carried out similar studies with other enzymatic protein biomolecules on electrode surfaces prepared from GQD precursors for electrochemical comparison, thus opening up potential sensing applications in medicine as well as bio-nanotechnology.

## 1. Introduction

Intense research into and development of graphene since its inception has been stimulated by the global demand for it in modern and future technologies [1,2]. Graphene is a two-dimensional honeycomb network of sp^2^ hybridized carbon (sp^2^ C) atoms having extraordinary physical and chemical properties (e.g., electrical conductivity, mechanical robustness, rich surface chemistry, reasonably good biocompatibility), attributed to its unique low-dimensional layered structure; it is studied for low power electronics, tunable optoelectronic and photonic devices, electrochemical energy conversion and storage systems, electrocatalysis, electrochemical sensing, biological sensing, theranostics, and in vitro and in vivo bioimaging [3,4,5,6]. While graphene is also a semimetal or zero-gap semiconductor, its bandgap can be tuned by its placement on a substrate, lateral dimension or nanosize, shape, thickness or number of layers, and fraction of sp^2^ C domains [7,8]. Graphene can also be altered through surface chemistry to give rise to various chemical forms and/or derivatives. Among others, graphene oxide (GO) [9,10] and reduced graphene oxide (rGO) [11] are emerging functional candidates facilitating tailored interfaces and tunable properties, especially when combined with other nanomaterials to expand the library of multifunctional materials [12,13,14]. GO is insulating due to the presence of saturated sp^3^-bonded carbon (sp^3^ C) bound to oxygen. It possess oxygenated covalent functional groups (COOH carboxyls and –ROOH epoxides at the edges and –OH hydroxyls on the surface) yielding remarkable mechanical strength, which is useful while assembling nanocomposites [15]. The reduced form, rGO, can be produced chemically, electrochemically, or thermally; it contains residual oxygen and permits semiconductor transition while offering tunable electrical conductivity and optical properties over several orders of magnitude depending on the carbon to oxygen ratio.

Carbon dots (C-dots) and graphene quantum dots (GQDs) are among the latest research frontiers within carbon-based nanomaterials [16,17,18,19,20]. They are attracting a great deal of scientific attention, particularly GQDs, due to their interesting excitation wavelength-dependent optical emission properties, namely photoluminescent excitation (PLE) properties [21,22], low toxicity, good photostability, chemical inertness, photo-electrochemical water splitting, and photocatalysis. It is noteworthy that their novel physicochemical and biological phenomena arise from the sp^2^ C nanosized core surrounded with edge plane functional moieties. On a fundamental level, zero-dimensional GQDs are produced from functionalized graphene (GO, rGO, few-layer graphene, or other 3D graphitic materials as precursors) by top-down synthetic approaches [23]. They normally exist in bi-layer structures with lateral dimension up to ~10 nm or smaller [24,25], with characteristics from the structure of graphene derivatives with quantum confinement size and edge plane sites effects resulting in opening the electronic bandgap for various nanoscale electronic and photonic applications [26,27,28]. The quantum confinement effect is crucial to discriminate a quantum material from its microscale graphene nanosheet and bulk graphite counterparts [29]. While enormous efforts have been made to reveal the origin of the PL emission of C-dots, only a few attempts have been carried out to investigate their roles and potential uses in electrochemistry and sensing applications [30,31]. This is despite the fact that numerous electrochemical studies have been performed on other nanoscale carbons including graphene-family nanomaterials, carbon nanotubes, porous carbon, carbon black, activated graphene, and activated carbon [32,33,34,35,36]. Likewise, in the field of electrochemical sensors and biosensors, there has been little attention to GQDs except for reporting on the fabrication of a specific biosensor that is based on the interaction between single-stranded DNA (ssDNA) [37] and GQDs. Recent interest has been in the development of novel or next-generation biosensors without using a reagent or a mediator but based on direct electron transfer of immobilized enzymes on carbon and other conducting substrates. Graphene and its derivatives have been readily used as electrochemical electrodes, exhibiting the ability to promote electron transfer of immobilized metalloproteins and enzymes [38] directly, and are used to construct third- or higher-generation electrochemical biosensors [39,40,41,42,43].

In this work, we report on the one-pot hydrothermal (water-based) and wet chemical solvothermal (organic solvent-based) syntheses of stable GQD colloidal dispersions of less than 10 nm size having functional edge plane sites derived from GO and rGO precursor suspensions. We explore the GQDs’ direct electrochemistry in the presence of a redox probe (ferrocene methanol; FcMeOH) and with those of GO and rGO precursors for direct electrochemical comparisons. We designed and investigated enzymatic adsorption capability of GQDs in promoting direct electron transfer, keeping in mind the electrochemical and electrocatalytic properties. Glucose oxidase (GO*_x_*) and horseradish peroxidase (HRP) enzymatic proteins and variants of graphene nanomaterials (GO and rGO) were selected. We used basal-plane pyrolytic graphite and glassy carbon (GC) electrodes modified with GQDs (GQD|GC), followed by activating with GO*_x_* (GO*_x_*-GQD|GC) and HRP (HRP-GQD|GC) using the drop cast method and air dried. The dispersions were characterized by UV-Visible and fluorescence emission spectroscopy and the electrodes were studied using cyclic voltammetry, differential pulse voltammetry, ac electrochemical impedance spectroscopy, and scanning electrochemical microscopy techniques. Subsequently, GO*_x_*-GQD|GC electrodes were examined as novel sensitive third-generation voltammetric and amperometric glucose biosensors for sensitive detection performance; the results are comparable to those of GO*_x_*-GO|GC and GO*_x_*-rGO|GC. The findings elucidate the significance of GQDs’ easy processability and sensitive detection of glucose, electrocatalytically highlighting the accessibility of sufficient hydrophilic edge plane sites density due to quantum size.

## 2. Materials and Methods

### 2.1. Preparation of Graphene Oxide, Reduced Graphene Oxide and Graphene Quantum Dots

**Figure 1:**
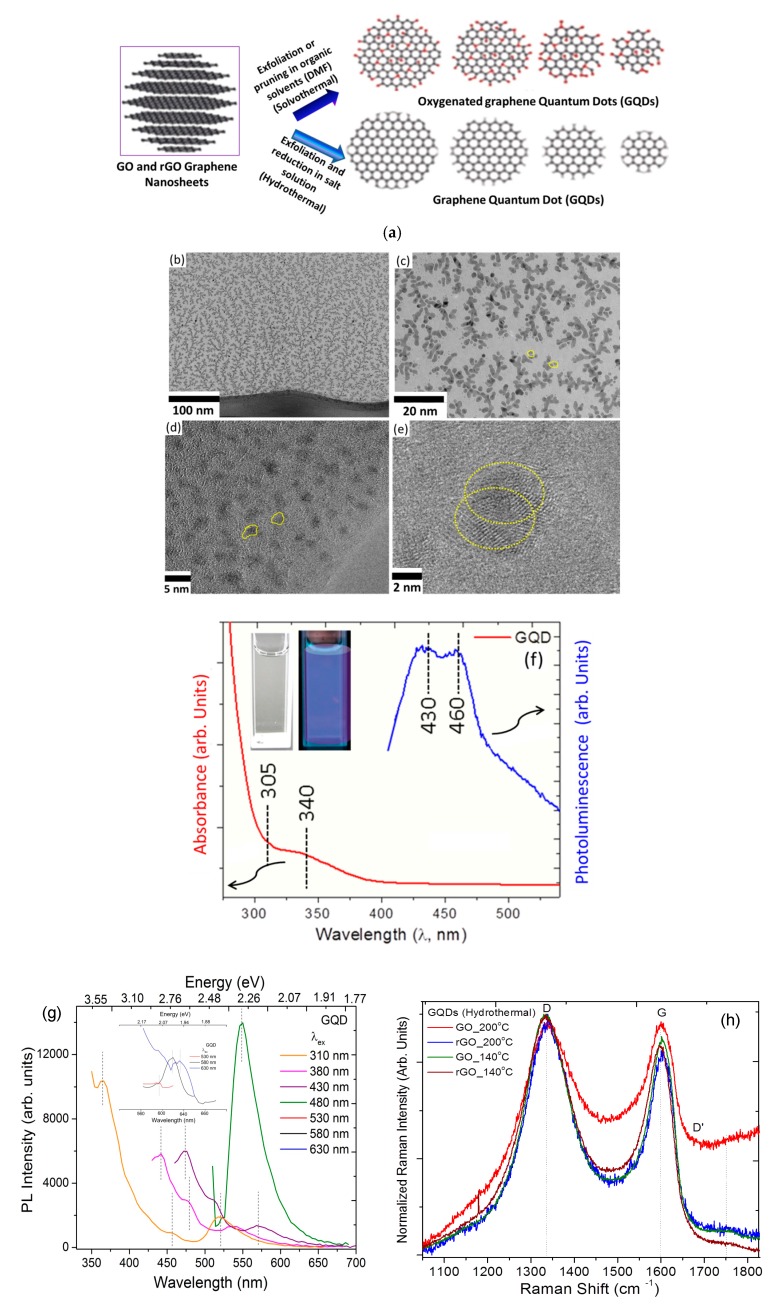
(**a**) Synthesis schemes for graphene quantum dots (GQDs) (top arrow; solvothermal) from exfoliation or pruning of GO in organic solvent (dimethylfluoride; DMF) and (bottom arrow; hydrothermal) exfoliation and reduction of GO (and rGO) in hydrothermal method resulting in GQDs of different sizes; (**b**–**d**) HR-TEM images of GQDs synthesized from scheme (a, top arrow) in different image resolution from 100 nm to 2 nm highlighting uniform dispersion and two isolated GQD overlapped depicting average size 5 nm (scale bars shown at the bottom of the images); (**f**) UV-Vis absorption (190–550 nm) and room temperature photoluminescence spectrum of GQD showing optical emission in blue-violet region (inset shows GQD vials without (clear) and with (blue violet) UV lamp); (**g**) photoluminescence excitation (PLE) spectra of GQDs; and (**h**) micro-Raman spectra of GQDs hydrothermal synthesized at two different temperatures using GO and rGO precursors.

Graphene oxide (GO) was prepared by a modified Hummer’s method, followed by chemical reduction using hydrazine monohydrate to produce reduced GO (rGO) [41]. We synthesized graphene quantum dots (GQDs) using a GO precursor and the solvothermal route [44] and hydrothermal method [45], wherein both GO and rGO were used (see Figure 1, panel a). The solvothermal preparation steps included 500 mg of GO added to 50 mL of *N*-*N* dimethylformamide (DMF) to produce 10 mg/mL concentrated GO/DMF suspension treated with ultrasonic dispersion (600 W) for 1 h, transferred into a 60-mL Teflon-lined stainless steel autoclave, and heated in a muffle furnace to 140 and 200 °C, both for 8 h. The final GQD/DMF product was obtained through vacuum filtration using a 0.2-μm micropore Nylon 66 membrane. The GQD/DMF suspension was roto-evaporated to remove DMF and to obtain GQDs, which were then re-dissolved in pure water and phosphate-buffered saline (PBS) to produce different suspensions. The GO/DMF of 0.5, 1, 5, 10, and 20 mg/mL were strictly controlled with 40%, 60%, and 80% ratios and reaction times of 8 h and 12 h. To select the optimal preparation conditions, the fluorescence quantum yield of different GOD samples was measured using fluorescence spectroscopy (excitation wavelength of 420 nm) with DMF as reference. For the hydrothermal method, GO powder was prepared as above and partially deoxidized in a tube furnace at <300 °C for 2 h at a heating rate of 5 °C·min^−^^1^ in an Ar atmosphere. The as-obtained thermally treated and chemically reduced GO nanosheet dispersions of 1.0 mg/mL concentration in deionized (DI) water were prepared by stirring for 8 h and mild ultrasonication (600 W, 35 kHz) for approximately 40 min. These dispersions were purified with microporous (0.2 μm) polytetrafluoroethylene (PTFE) membrane and re-dispersed in DI water. Then the suspensions were heated at 200 °C for 10 h in a Teflon-lined stainless steel autoclave. The resulting black suspensions were filtered with 0.2 μm PTFE membrane and a dark brown filtered solution was obtained. To remove larger graphene nanoparticles, the colloidal solution was dialyzed retaining molecular weight, 3500 Da overnight. The same can also be obtained by filtration and centrifugation methods. The GQDs obtained from these two precursors showed stability over six months. These dispersions were drop cast followed by air drying on commercial indium tin oxide (ITO) coated glass substrates of 1 × 4 cm^2^ size and on glassy carbon (GC) electrodes for the various electroanalytical studies mentioned below.

### 2.2. Preparation of GQD-Modified and GO_x_-Immobilized Glassy Carbon Electrodes

Glucose oxidase, GO*_x_* (from *Aspergillus niger*, E.C.1.13.4) was obtained from Sigma-Aldrich (St. Louis, MO, USA) and the stock solution was prepared in 50 mM phosphate buffer solution (PBS) pH 7.8 and stored at 4 °C. d(+)-glucose, ferrocene methanol (FcMeOH) and glassy carbon (GC) 4 mm diameter rods were purchased from Merck Chemicals (Kenilworth, NJ, USA) and Alibaba Co. (Hangzhou, China), respectively. All other chemicals were of analytical grade and used without further purification.

Due to the unique properties of GCs—good electrical conductivity, renewable/reusable electrode surface, mesoscale porosity, and cost effectiveness—they were used to immobilize graphene nanosheets, graphene quantum dots, enzymatic proteins, and combinations thereof for electrochemical and biosensing investigations. The as-received GCs were shined with alumina paste and a polishing cloth, and subsequently washed and rinsed several times with DI water. The GQD solution (50 μL), GO and rGO (both 70 μL) were placed thrice using a Hamilton syringe on GC substrates and dried overnight at room temperature to obtain homogeneous GQD|GC, GO|GC and rGO|GC samples. To activate these electrodes, pre-treatment was performed electrostatically at +1.5 V for 100 s. Finally, 20–25 μL of GO*_x_*, Mb (Myoglobin), Cyt *c* (Cytochrome *c*), and HRP (all 2.0 mg/mL concentration in DI water) were immobilized via physical adsorption using a Hamilton syringe to prepare GO*_x_*-GQD|GC, GO*_x_*-GO|GC, GO*_x_*-rGO|GC, Mb-GQD|GC, Cyt *c*-GQD|GC, HRP-GQD|GC electrodes. For slow evaporation of water and also for uniform film formation, the electrodes were covered with a petri dish. All of the modified electrodes were stored at 4 °C in refrigerator when not in use.

### 2.3. Sample Characterization

#### 2.3.1. Structural and Optical Characterization

All the samples were characterized to obtain surface morphology at nanoscale, crystallinity, optical and lattice vibration properties. Samples for high-resolution transmission electron microscopy (HR-TEM) were prepared by placing two drops of GQDs on commercial Cu grids coated with lacey carbon (Ted Pella Inc., Redding, CA, USA) and allowing them to air dry. They were taken using a JEOL instrument (Model 1400 Plus, Peabody, MA, USA) operating in Cryo-EM (Pleasanton, CA, USA) and SAED (Selected-Area Electron Diffraction, Jacksonville, FL, USA) modes at 100 or 200 kV and 1 nA with a Be specimen holder and a Gresham SiLi detector with Moxtek AP3.3 window. TEM measurements provided size distribution, intrinsic microstructure, and lattice spacing. The measurements were also performed in the STEM mode using a nanoprobe with probe size of ~1 nm. STEM images were collected with a Fishione HAADF (High-Angle Annular Dark-Field, Pleasanton, CA, USA) detector and EDX (Energy Dispersive Spectroscopy, Pleasanton, CA, USA) signals were measured using an EDX detector yielding C/O ratio of 8:1 for both GQDs prepared using GO and rGO. Interestingly, GO is reduced while undergoing hydrothermal treatment. The optical (UV-Vis absorption and fluorescence) spectroscopy measurements were taken using a BioTek spectrometer (Model Synergy H1 Multi-mode Reader, Winooski, VT, USA) equipped with a xenon lamp as broadband excitation source. For fluorescence measurements, the excitation wavelength *λ*_ex_ = 370 nm and spectral window of 350–550 nm was used with wavelength interval 0.5 nm and for UV-Vis the absorption spectroscopy is measured between 210 and 550 nm in interval of 1 nm. All the measurements were carried out at room temperature (~298 K). Raman spectroscopy was carried out to determine the lattice vibration structure at various points on GQDs and other samples. The Raman spectra were recorded using a micro-Raman spectrometer (Model InVia Renishaw plc, Gloucestershire, UK) equipped with laser excitation wavelength 633 nm (*E*_L_ = 1.92 eV) and ~1–2 mW power incident at the sample, with edge filters cutting at ~100 cm^−1^ and an objective lens of 50× providing spot size ~2 μm. The scattered light from the sample is collected in backscattering geometry, transmitted by a beam splitter, and detected by a CCD camera. Extreme care was taken to avoid sample damage caused by laser-induced thermal degradation and therefore 5% or 10% light intensity was used to obtain spectra with acquisition time per pixel ranging from 60 s to a few minutes, though it was increased to 300 s to optimize the signal-to-noise ratio. Raman shift ranged from 1100 to 3200 cm^−1^ with a spectral resolution of 1 cm^−1^.

#### 2.3.2. Electrochemical Properties

A custom designed three-electrode electrochemical cell was used with a bi-potentiostat electrochemical workstation (CH Inc. Model 920D, Austin, TX, USA) for electrochemical measurements in cyclic voltammetry (CV), differential pulse voltammetry (DPV), and *ac* electrochemical impedance spectroscopy (ac EIS) modes, where a saturated Ag/AgCl (3M KCl) electrode and Pt wire (3 mm diameter) were used as reference and counter electrodes, respectively. These techniques were used to investigate electrode kinetics and in turn assess the working electrodes’ performance. The GQDs and other graphene-based samples were used as working electrodes characterized in 0.1 M PBS electrolyte within the potential range −0.9 and +0.9 V at various scan rates *v* = 5, 10, 20, 50, 100, 200, 300, 400, 500 mV/s in CV mode. The *ac* EIS measurements were conducted in the frequency range 10 mHz–98 kHz at +0.3 V superimposed with alternating current voltage amplitude 10 mV. For GO*_x_* sensing, the CV measurements were carried out from −0.8 to 0.2 V at a 20 mV/s scan rate with successive addition of glucose concentration (0.5, 1, 1.5, 2.0, 2.5 and 3 mM) for GO*_x_*–GQD|GC. The differential pulse voltammetry (DPV) was also used for enhanced sensitivity between potential range −0.8 and +0.8 V at *V*_amp_ = 25 mV. Amperometry (*i*-*t*) monitored the current with discrete additions of 10 and 100 μM glucose at a constant potential of −0.4 V in 0.1 M PBS, pH 7.4 at intervals of 50 s until 1200 s on various electroanalytical platforms [GO*_x_*-(GO, rGO, GQD)|GC]. Scanning electrochemical microscopy (SECM) was employed to gain further insights into the electrode/electrolyte physicochemical interfacial processes and quantify the associated parameters [44]. Briefly, SECM measurements are carried out in cyclic voltammetry, probe approach in negative feedback modes using the same bi-potentiostat mentioned above. This technique uses a Pt microelectrode (~5 μm) tip as working electrode 1 with 5 mM ferrocene methanol (FcMeOH) redox probe in support electrolyte 0.1 M PBS. FcMeOH has a standard potential *E*^0^ = +0.21 V versus Ag/AgCl. The Pt tip electrode was held at a potential of *V*_t_ = +0.5 V to ensure complete diffusion-limited oxidation of Fe(II) species originally present in the electrolyte solution to Fe(III) with substrates as working electrode 2. For SECM imaging method, the electrodes (tip and substrate) were biased at *V*_t_ = +0.5 V and *V*_S_ = −0.4 V, respectively. The tip was rastered over the working electrode 2 (GQD, GO*_x_*-GQD, HRP-GQD, Mb-GQD, Cyt *c*-GQD, Cyt *c*-GO and GO*_x_*-rGO) surface area (500 μm × 500 μm) kept at a constant tip–substrate separation ≤8–10 μm to generate a feedback image with an approximate resolution of tip radius with a sub-nanoampere level of current sensitivity. These studies were performed several times on multiple electrodes and the results were reproducible within <98% for all of the electrodes.

## 3. Results and Discussion

### 3.1. Microscopic Structure and Physical Properties

Figure 1 (panel b–e) shows high-resolution transmission electron microscopy images revealing the surface morphology, monodispersity and average particle size (~5–6 nm) of the GQDs sheets with interplanar spacing, d_002_ ~0.345 nm (ca. parent graphite, 0.34 nm); the latter is derived from the presence of Moiré fringes. Structural order is evident from the lattice fringe patterns associated with crystalline graphene sheets. The GQDs contain similar types of oxygenated functional groups as their precursors, including carbonyl (–C=O), carboxyl (–COOH), epoxy (–O–), and hydroxyl (–OH), distributed at the multilayered terrace or edge planes and preferential reduction produces reduced GQDs, where the interplanar spacing is comparable to traditional graphene. High-resolution TEM images also show partially wrinkled GQD layered sheets with sub-micro-level porosity beneficial to mass transfer of electroactive species, stabilization, and effective immobilization of dispersed biological molecules. The energy-dispersive X-ray spectroscopy (EDX) spectra (not shown) yielded C to O ratios ranging between 55.34 and 69.27 at. %, as anticipated.

UV-Vis absorption and photoluminescence spectroscopy revealed the characteristic bands associated with as-prepared GQDs (Figure 1f). Broad weaker absorption bands at ~305 nm and at ~340 nm and photoluminescence peaks at 430 (2.87 eV) and 460 nm (2.68 eV), excited at 370 nm (3.34 eV), are characteristic of apparently blue-violet GQDs according to various reports [26,43]. The absorption band at ~278 nm corresponds to π (bonding)–π* (antibonding) transition (characteristic of natural π-conjugated graphene sheets) of aromatic sp^2^ C domains. Like most of the work reported, as-synthesized GQDs possess excitation wavelength-dependent PL (PLE) showed in Figure 1g, where the spectral maxima shift with excitation energy. In addition, the peaks at 305 nm and 340 nm do not shift with excitation energy and are independent of reaction duration, thus the size of GQDs does not matter. Therefore, the variable PL maxima and the invariable peak at ~340 nm suggest multiple emissive mechanisms responsible for the overall emission from GQDs. It is proposed that the blue shift of PL maxima is due to quantum confinement of excitons, according to which the smaller the GQDs size, the wider the bandgap and the higher the emission energy [45,46]. Moreover, the oxygenated functional groups combined with atomic scale defects produce irregularly hybridized π states, which causes so-called energy states induced by structural deformation (*ESiD*). These *ESiD* possess energy levels in-between the HOMO/LUMO (Highly Occupied Molecular Orbitals)/Lowest Occupied Molecular Orbits) gap that serve as intermediate or mid-gap states between bonding and anti-bonding states. The micro-Raman spectra are displayed in Figure 1h for GQDs prepared with GO and rGO at 140 and 200 °C. For a realistic comparison, the spectra are normalized to G band. Raman spectroscopy shows prominent signatures corresponding to the defects in the graphene basal plane (i.e., D band at ~1350 cm^-1^) and the characteristic peak associated with pristine graphitic or sp^2^-bonded carbon (sp^2^ C) materials (in-plane stretching or tangential G band at ~1590 cm^−1^), respectively [47,48]. Alternatively, they correspond to the breathing mode of κ-point phonons of A_1g_ symmetry and the first-order scattering of E_2g_ phonons of graphene, respectively. While the relatively narrow G band indicates structural or crystalline order, the presence of a finite D band originates from the large density of defects in GQDs sheets. It is worth mentioning that no evidence about the disorder-induced signal (D’ band) occurring at 1620 cm^−^^1^ has been offered. The intensity ratio of D to G band (*I*_D_/*I*_G_) is a semi-quantitative measure of structural order, i.e., in-plane sp^2^ C clustering and a larger ratio mean smaller sp^2^ C domains. All of the microscopic structural characterization results indicate that high crystalline quality GQDs are synthesized successfully.

**Figure 2:**
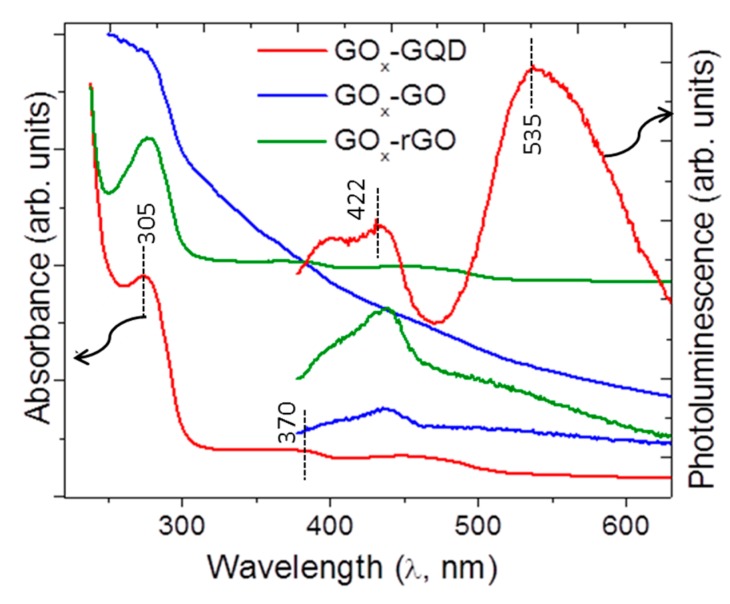
UV-Vis absorption and photoluminescence spectra of enzymatic GO*_x_* protein in the presence of water-dispersed GQD, GO, and rGO, depicting characteristic peaks.

The UV-visible absorption spectroscopy is a useful tool that can reveal changes in proteins’ secondary structure upon adsorption on graphene nanomaterials affecting their static and dynamic activities. For details on the absorption spectra of metalloproteins and HRP enzymatic protein and their interaction with graphene substrates, refer to our earlier work [38]. Briefly, Cyt *c* has an absorption peak at ~280 nm arising from the conjugated double bond in the residue of aromatic amino acids (tyrosine, tryptophan, and phenylamine), common to all proteins. In particular, metalloproteins have characteristic absorption bands at about 408 nm (Soret band) and 527 nm (Q-band), both produced by the chromophore of porphyrin ring; the position and intensity changes are reflective of conformation or asymmetry and interaction with graphene-family nanomaterials. Figure 2 shows the UV-Vis absorption and photoluminescence (PL) spectra of enzymatic GO*_x_* protein in the presence of water-dispersed GQDs, with GO and rGO displaying a characteristic peak around 270 cm (π–π* transition), revealing π-conjugation of graphene sheets. Apparently GO*_x_* has a sharp absorption peak at 277 nm along with a pair of peaks at 373 and 451 nm (and corresponding 422 and 535 nm emission peaks in PL spectra), consistent with previous reports [49]. It is evident from the absorption peaks that GO*_x_* is adsorbed on GQDs, GO, and rGO surfaces. The peak at ~370 nm is the absorption of the conjugated double bond in the residue of aromatic amino acids (tyrosine, tryptophan, and phenylamine) in the proteins, which is common to most of the metallo- and enzymatic proteins. The absorption spectroscopy can also reveal changes in secondary structure that may occur upon adsorption on graphene substrates and consequently affect their static and dynamic activities.

### 3.2. Cyclic Voltammetry, Differential Pulse Voltammetry, and ac Electrochemical Impedance Spectroscopy

**Figure 3:**
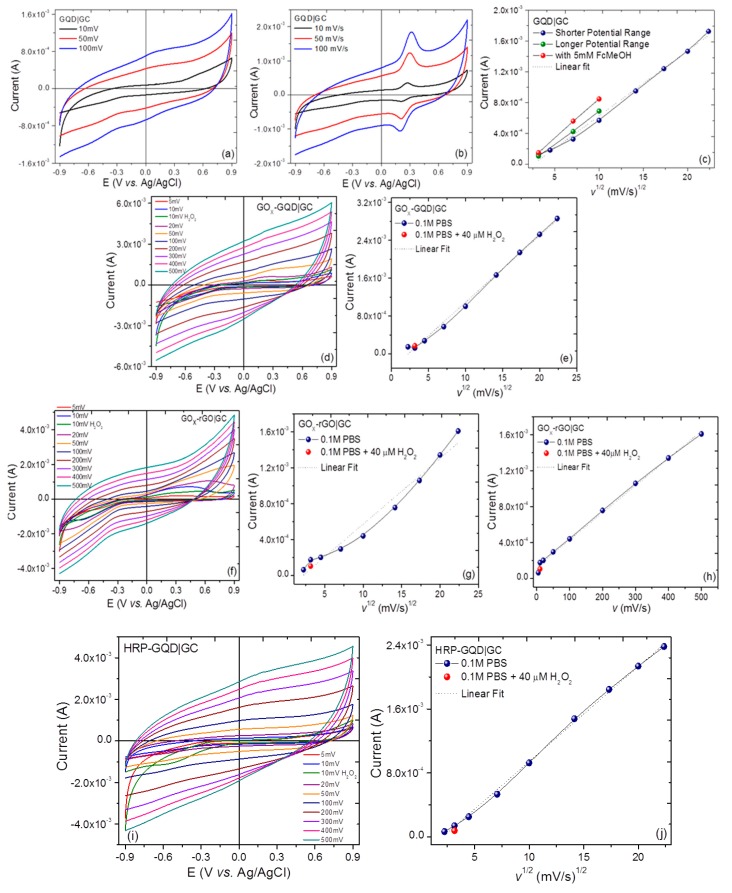

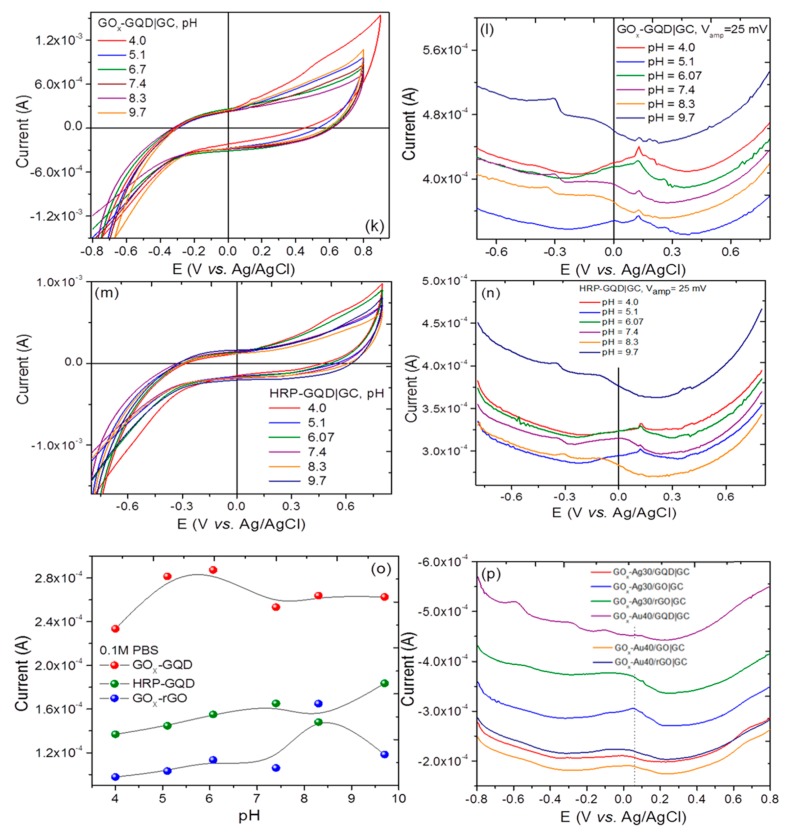
Cyclic voltammograms (CVs) of GQD immobilized on glassy carbon-GQD|GC with scan rate (10, 50 and 100 mV/s) in (**a**) in 0.1 M PBS (phosphate buffer solution) electrolyte pH 7.4 (**b**) in 0.1 M PBS electrolyte with 5 mM FcMeOH (**c**) variation of peak current with scan rate square root. (**d**,**f**) CV profiles and (**e**,**g**,**h**) variation of current with scan rate and square root scan rate (5, 10, 20, 50,100, 200, 300, 400 and 500 mV/s) in 0.1 M PBS and at 10 mV/s with 40 μM H_2_O_2_ adsorbed on GQD|GC and GQD|GC, respectively. Also shown are (**i**) CV profiles and (**j**) variation of peak current with scan rate square root for native horseradish peroxidase (HRP) adsorbed on GQD|GC for comparison with GO*_x_* and (**k**,**m**) CV and (**l**,**n**) differential pulse voltammetry (DPV) profiles for GO*_x_* and HRP adsorbed on GQD|GC with different pH (4.0, 5.1, 6.07, 7.4, 8.3, 9.7) of PBS electrolyte and (**o**) variation of peak current for GO*_x_*, HRP adsorbed on GQD|GC and GO*_x_* on rGO|GC substrates. (**p**) DPV profiles for GO*_x_* adsorbed on Ag30/(GQD, GO, rGO)|GC and Au40/(GQD, GO, rGO)|GC. All of the potentials are with respect to Ag/AgCl reference electrode for CV profiles and with varying pH they are measured at 20 mV s^−1^ scan rate.

Following structural and bonding characterization, we now focus on the electrochemical properties of as-synthesized GQDs as well as GO and rGO for comparison. With several advantages of electrochemical properties similar to graphene and emergent functionalized graphene nanomaterials, GQDs are also applicable as novel electrodes in the field of direct electrochemistry and electrochemical biosensing. Moreover, the tunable size (hence tunable energy gap) of GQDs allow them to act as multivalent redox species studied using cyclic voltammetry (CV) and differential pulse voltammetry (DPV), present exciting opportunities. Figure 3 shows cyclic voltammograms (CVs) of GQDs-modified GC electrodes in 100 mM PBS electrolyte (pH 7.4) between −0.9 and 0.9 V (panel a). Qualitatively, the CV loop was sufficiently rectangular, desirable for a supercapacitor, and the rate capability was examined at various scan rates (10, 50, and 100 mV/s); the rectangular area increased with increased scan rate, as expected. The GQDs generated weaker anodic/cathodic peaks at 0.275/−0.28 V (vs. Ag/AgCl), which becomes pronounced with the addition of FcMeOH (ferrocene methanol) redox mediator (Figure 3b). The CV shows reversible redox peaks with peak-to-peak separation ∆E_p-p_ ~945 mV. Figure 3c shows the variation of maximum current with square root of scan rate (*v*^1/2^) and a quasi-linear behavior (heterogeneous), especially at higher scan rates due to diffusion-limited (mass transport) phenomena attributed to surface and edge oxygenated functional groups. The magnitude of the observed current is governed by the Randles–Ševćik equation for a reversible transfer process, $I_{rev}=0.446FAC{\left(FDv/\mathrm{RT}\right)}^{0.5}$ or $I_{rev}=(2.69*10^{5})n^{3/2}ACD^{1/2}v^{1/2}$, where *A* is the geometric area of the electrode (cm^2^), F is the Faraday Constant (C mol^−1^), *D* is the diffusion coefficient (cm^2^ s^−1^), *C* is the concentration (mol/cm^3^), *v* is the scan rate (V s^−1^), R and T are usual constants, and *n* is the total number of electrons transferred in the electrochemical process [3,34,44]. The analysis of current behavior helped to determine *D*, which is 3 × 10^−8^ m^2^ s^−1^. The heterogeneous electron transfer rates (*k*_ET_) of the GQDs are compared with the bare GC electrode surface using redox a probe. FcMeOH was determined to be 1.45 × 10^−7^ cm s^−1^ following Laviron’s theory [50]. The GQD generated larger peak current signals than the bare GC electrode surface and therefore was deemed significant for sensing biological molecules electrochemically. FcMeOH (and potassium ferricyanide; K_3_Fe(CN)_6_)) are surface-sensitive redox probes that enable us to distinguish among different nanoscale carbons including graphene surfaces, which is usually not possible with an outer-sphere (electron transfer reaction that involves no bond breaking or bond formation process during redox processes) ruthenium hexamine (Ru(NH_3_)_6_ redox probe [51]. We also determined the diffusion coefficients for GO and rGO to be 1.5 × 10^−9^ and 0.9 × 10^−9^ m^2^ s^−1^, respectively (not shown) [34]. An increase in *D* values for GQDs as opposed to GO and rGO nanosheets is attributed to quantum size confinement, thus permitting access to numerous hydrophilic edges as well as basal plane defects and functional groups.

Following this study, the electrochemistry of glucose oxidase (GO*_x_*) immobilized on GQD|GC electrodes was investigated in Ar-saturated 100 mM PBS (pH 7.4) at several scan rates (5, 10, 20, 50, 100, 200, 300, 400, 500, with 40 μM H_2_O_2_) mV s^−1^ in CV mode (see Figure 3d). With increasing scan rate both redox peak current magnitude and peak-to-peak separation (∆E_p-p_) increase. This behavior is characteristic of typical surface sensitive dynamic physicochemical process (electrode kinetics) where cathodic and anodic peak currents are of a similar magnitude with a ratio of about one. The CV plots for GO_x_ with peak potential at −0.252/+0.288 V and peak-to-peak separation (∆E_p-p_) 66 mV represents quasi-irreversible electron transfer process of redox active center i.e., Flavin adenine dinucleotide, FAD in GO*_x_* [52,53]. The presence of 40 μM H_2_O_2_ did not show a noticeable difference in contrast to HRP (see Figure 3i, CV at 10 mV/s scan rate), and the behavior of the current is analyzed in Figure 3j. Similar measurements were carried out on GO*_x_*-rGO|GC (Figure 3f) and GO*_x_*-GO|GC (not shown). The peak current is plotted as a function of *v*^1/2^ for GO*_x_* immobilized on GQD and rGO (Figure 3e, g and h) showing linear behavior for GQDs and marginal deviation from linearity for rGO (and GO, not shown) samples. We deduced the D values in the presence of GO*_x_* following the same approach as above, which are 1.45 × 10^−9^, 2.78 × 10^−9^ and 5.0 × 10^−8^ m^2^ s^−1^ for GO, rGO and GQD, respectively. It is apparent that GQDs demonstrate reasonably good electrocatalytic properties toward GO*_x_* (marginally good for HRP), with comparable results for GO and rGO precursors. We attempted to determine *k*_ET_, which is significantly higher for GO*_x_*-GQD than rGO and GO precursors. The integration of CV peaks provides the total charge (*Q*) passed through the electrodes for a redox reaction of electroactive enzymes. The surface coverage (*Γ*) of electroactive GO*_x_* can also be determined by following *Γ* = *Q*/*n*F*A*, where *n* is the number of electrons transferred (2), F is the Faraday constant, and *A* is the electrode area (0.27 cm^2^). The estimated values are 3.9 × 10^−9^, 0.7 × 10^−9^, and 2.35 × 10^−10^ mol cm^−2^ for GQDs, rGO, and GO, respectively. These values are larger than those adsorbed on a bare GC electrode (2.86 × 10^−12^ mol cm^−2^), reflective of efficient surface coverage with effective surface adsorption. Next, we studied the influence of pH on electrochemical behavior. It is well established that direct electrochemistry of GO*_x_* is a two-electron coupled with two-proton reaction, undergoing a redox reaction: GO*_x_* (FAD) + 2e^−^ +2H^+^ ←→ GO*_x_* (FADH_2_). Figure 3k–n shows cyclic and differential pulse voltammograms for GO*_x_*-GQD|GC and HRP-GQD|GC at a scan rate of 20 mV/s displaying reversible redox peaks at pH ranging between 4 and 10. The peak potential shifts to the negative direction when the electrolyte solution pH changes from 4 to 9.7. The slope of −52.6 mV/pH is comparable to the reported (−55.7 mV/pH) and theoretical values (−58.5 mV/pH) [54]. The marginal discrepancy is supposedly due to the size of the GQDs and the higher accessibility and density of hydrophilic edge functional groups. Taken together, the response of GQDs to GO*_x_* is substantially higher than rGO, GO and HRP on GQDs (see Figure 3o). Motivated by plasmon-assisted enhanced electrocatalytic activities [55,56], where covalently assembled GQDs with enriched periphery carboxylic groups on gold electrode showed high peroxidase activity and promising electrocatalytic properties toward H_2_O_2_ decomposition, we carried out DPV measurements (see Figure 3p) on GO*_x_*-GQDs and GO*_x_*-rGO decorated with silver (Ag) and gold (Au) nanoparticles of average size 30 and 40 nm, respectively. The signal was clearly improved with prominent peaks corresponding to GO*_x_* activity showing the role of noble metal nanoparticles inducing localized re-hybridized orbitals favorable for electrochemical activity [57]. 

**Figure 4:**
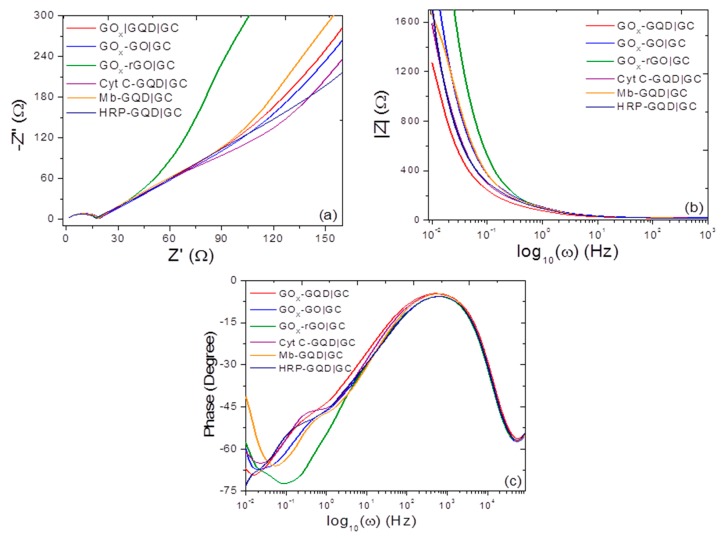
Electrochemical impedance spectroscopy (EIS) spectra of (**a**) Nyquist plot (−Z” versus Z’); (**b**) magnitude of Z versus log of frequency; and (**c**) phase behavior for GO*_x_* adsorbed on GQD|GC, GO|GC, rGO|GC, native cytochrome (Cyt *c*) adsorbed on rGO|GC, native myoglobin (Mb) and HRP adsorbed on GQD|GC.

The electrochemical impedance spectroscopy (EIS) technique is helpful in providing further information about the electrode kinetics, to quantify electronic and ionic contributions and diffusive behavior. For instance, a typical impedance spectrum showing a semicircle at higher frequencies and a straight line at lower frequencies correspond to the electron-transfer (charge transfer) limited process and the diffusion (mass) process, respectively. The charge transfer resistance (*R*_ct_) can be determined from the radius of the semicircle under ideal conditions. Here, we investigated the surface of GO*_x_*-GQD, GO*_x_*-GO, and GO*_x_*-rGO and compared them with the HRP-GQD, Cyt *c*-GQD, and Mb-GQD electrodes. Figure 4a shows Nyquist (−Z” versus Z’) plots and Figure 4b shows Bode plots (|Z| versus log of frequency). They exhibit a straight sloping line (i.e., ion diffusion and mass transport in bulk electrolyte) at smaller frequencies, resulting from the electrolyte to the electrode surface (i.e., grain boundary interfaces and diffusive resistance), and the semicircle (or arc) at the high-frequency region corresponds to the charge transfer resistance (*R*_ct_) caused by the pseudocapacitive or Faradaic reactions of electrodes. The increasing slope indicates a capacitive nature that is related to the charging mechanism characteristic of mesoporous (hierarchical porosity) electrodes with a three-dimensional network. The phase difference of almost 70 degrees (see Figure 4c) at higher frequencies is in fact directly correlated with the semicircle capacitive nature in Nyquist plots (Figure 4a). The relatively lower *R*_ct_ values (~5–6 Ω) for all electrodes indicate an enhancement in the electronic and ionic conductivities, facilitating rapid electron transfer between the modified GC electrodes and the redox probe molecule. However, a marginal difference in *R*_ct_ is due to negatively charged –COOH functional groups that may inhibit full penetration at the electrode/electrolyte interface. Also, the GQDs and other functionalized graphene are semiconducting, having lower conductivity as opposed to traditional mono- or multi-layered graphene, which is semimetal. Thus it is possible that the energy bandgap and *R*_ct_ of tunable sized GQDs may be directly proportional, within certain limits.

### 3.3. Voltammetric and Amperometric Glucose Sensing

**Scheme 1:**
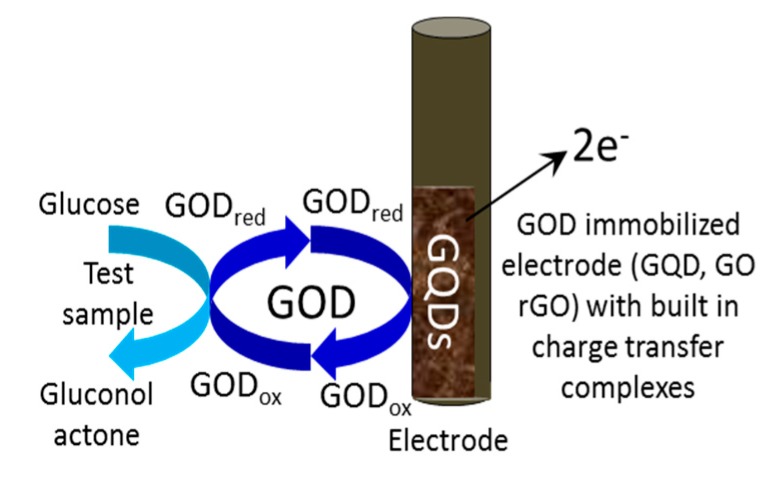
Schematic of enzymatic glucose sensing principle on immobilized GQDs.

**Figure 5:**
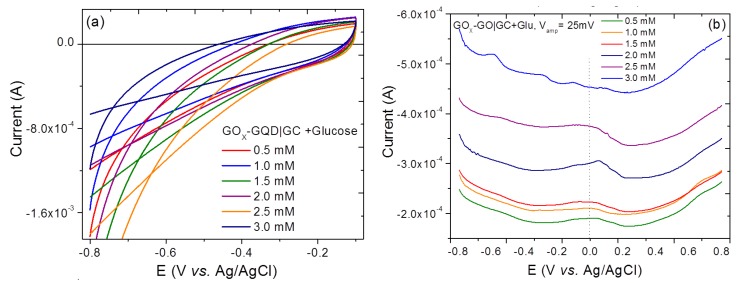

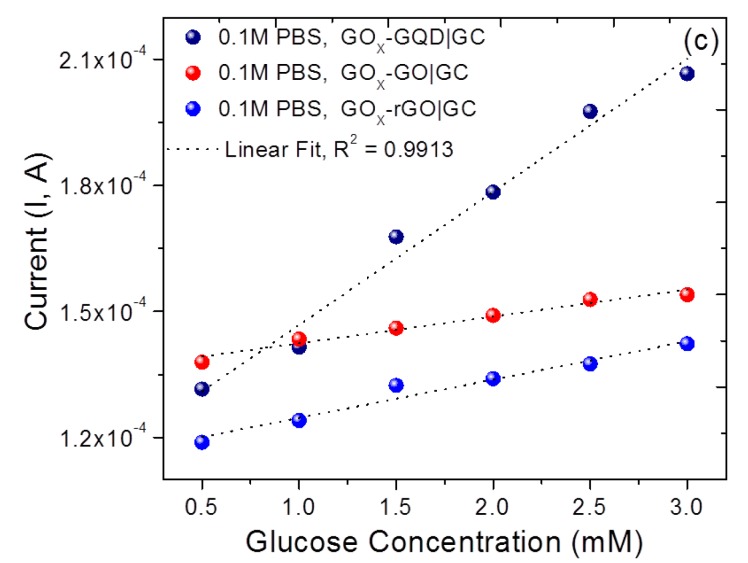
(**a**) Cyclic voltammograms; (**b**) DPV profiles; and (**c**) variation of peak current with varying glucose concentration (0.5, 1, 1.5, 2.0, 2.5 and 3 mM) for GO*_x_*–GQD|GC. The CV profile with glucose is measured at 20 mV/s scan rate. All of the potentials are with respect to the Ag/AgCl reference electrode.

**Figure 6:**
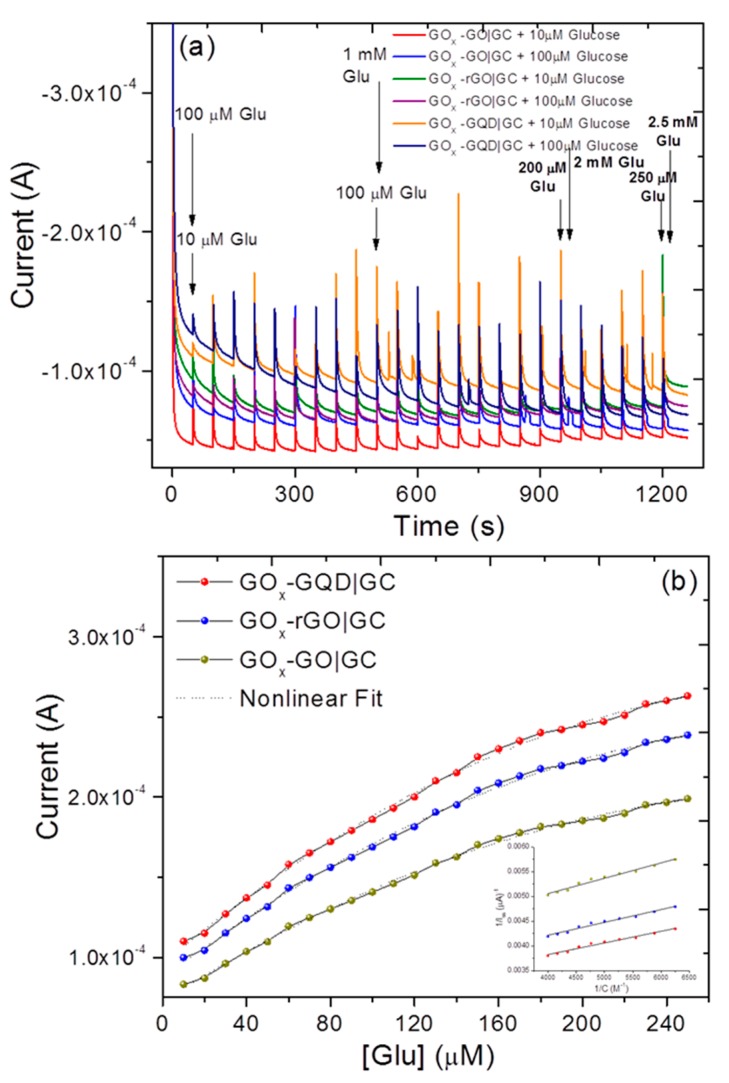
(**a**) Representative amperometric response (*i*-*t*) to successive addition of 10 μM and 100 μM glucose step under conditions: −0.4 V constant potential, 0.1 M PBS, pH 7.4 in interval of 50 s until 1200 s on various electroanalytical platforms [GO*_x_*-(GO, rGO, GQD)|GC] and (**b**) chronoamperometric current versus glucose concentration (100 mM) amperograms obtained from (**a**). Inset in (**b**) shows Lineweaver–Burk plot for *K*_m_ value determination.

In this section, we investigate the biocatalytic activity of GO*_x_*-GQD|GC toward reduction of oxygen, for which the process is expressed as: GO*_x_*(FADH_2_) + O_2_ → GO*_x_*(FAD) + H_2_O_2_. Scheme 1 shows the principle of enzymatic glucose sensing, which does not require a mediator and is in fact governed by the presence of inbuilt charge complexes on the electrode, responsible for electrochemical glucose sensing. The cyclic and differential pulse voltammograms shown in Figure 5a,b, respectively, are in presence of 0.1 M PBS with glucose from 0.5 to 3 mM concentration. While the redox peaks are almost absent in CV profiles, the DPV curves exhibit a decline in reduction peak with increasing glucose concentration, ascribed to the decrease in oxygen on the electrode surface caused by the enzyme-catalyzed reaction between the oxidized form of GO*_x_* (FAD) and glucose following the Glucose + GO*_x_*(FAD) → gluconolactone (C_6_H_10_O_6_) + GO*_x_*(FADH_2_) reaction [58]. The current decrease is quasi-linearly proportional to glucose addition, suggestive of determination of sensitivity of glucose concentration. To further elucidate the relationship between electrocatalytic reduction current and glucose concentration, the chronoamperometry technique was performed at constant potential −0.4 V in 0.1 M PBS, pH 7.4 (see Figure 6a). The steady-state current obtained on GO*_x_*-GQD|GC decreased during successive addition of glucose concentration (Figure 6a,b). The electrocatalytic response was faster as the biosensor achieved more than 97% of maximum response less than 4 s when glucose was added into the electrolyte solution. The current response versus glucose concentration was linear over a wide concentration range (10–250 μM), exhibiting 0.00769 μA·μM^−1^·cm^−2^ sensitivity, 0.998 correlation coefficient, and 1.35 μM limit of detection (LOD). The parameters such as sensitivity, linear range, and limit of detection for the developed GQD-based biosensor are much improved (by an order of magnitude) as compared to those of reported values 0.085 μA μM^−1^ cm^−2^ [53] and rGO (0.025 μA μM^−1^ cm^−2^) and GO (0.064 μA μM^−1^ cm^−2^). When the concentration was higher than 1.12 mM, the electrode response deviated from linearity and a plateau was observed, characteristic of Michaelis–Menten kinetics [58]. The Michaelis–Menten constant is an important parameter for enzyme–substrate reaction kinetics that is obtained from the Lineweaver–Burk equation following [58]: $\frac{1}{I_{SS}}=\frac{1}{I_{\max}}+\frac{K_{m}}{I_{\max}}\frac{1}{C}$, where *C* is the concentration of glucose, *I*_SS_ is the steady-state catalytic current (background corrected), and *I*_max_ is the maximum catalytic current. *K*_m_ is obtained by the slope analysis and the intercept of reciprocal plots of the *I*_SS_ versus inverse glucose concentration (see Figure 6b, inset) amounted to 0.54 mM, comparable to or better than those for rGO (0.66 mM) and GO (0.75 mM) as well as currently reported glucose biosensors [53]. The lower *K*_m_ refers to retention of GO*_x_* native structure for GQDs-modified GC electrodes, resulting in higher affinity and electrocatalytic activity of GO*_x_* toward glucose in an enzymatic reaction.

### 3.4. Scanning Electrochemical Microscopy

**Figure 7:**
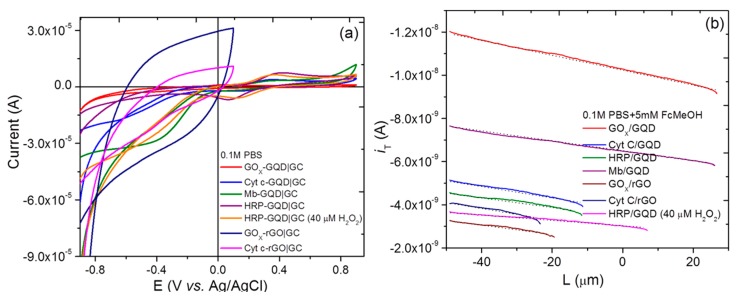
(**a**) Representative cyclic voltammograms (CVs) in microscale electrode (convergent diffusion) configuration; (**b**) variation of heterogeneous diffusion-controlled tip electrode current (*i_T_*), which becomes asymptotic with increasing tip–substrate distance (L) including probe approach curves for GO*_x_*-GQD|GC, Cyt *c*-GQD|GC, Mb-GQD|GC, HRP-GQD|GC, HRP-GQD|GC (with 40 μM H_2_O_2_), GO*_x_*-rGO, and Cyt *c*-rGO|GC. The curves are indicative of semiconducting (or insulating) behavior at the solid/liquid interface in redox mediator 5 mM FcMeOH (ferrocene methanol) in support electrolyte 0.1 M PBS, conditions: tip voltage *V*_t_ = +0.5 V and substrate voltage *V*_s_ = −0.4 V.

The above findings suggest that the electrocatalysis is related to the density of electroactive species and edge plane sites, which underscores the significance of surface morphology while rationally designing efficient glucose biosensors. Therefore, it is conceivable to investigate the nature of interfacial processes at electrode/electrolyte (solid/liquid) interfaces. By detecting redox reactions occurring in a small region in close proximity to electrode surface (probe approach and feedback modes), scanning electrochemical microscopy (SECM) is used to obtain local quantitative information of reaction rates, to probe charge transfer and ion transport dynamics, and to determine electroactive adsorption sites density [44,59]. SECM monitors electrochemical (ionic and electronic) currents to be mapped and chemical reactivity images correlating with the morphological structure as opposed to other scanning probe microscopy [44,60,61,62]. Figure 7a shows CV curves at a scan rate of 20 mV/s measured in a single-compartment, three-electrode electrochemical cell with an SECM Pt tip (micro-electrode configuration) for GO*_x_*-GQD|GC, GO*_x_*-rGO|GC, GO*_x_*-GO|GC, Cyt *c*-GQD|GC, HRP-GQD|GC, and Mb-GQD|GC electrodes. In particular, all the electrodes displayed a characteristic pair of redox peaks, albeit broad. In traditional electrochemistry, the reactions occur across the entire electrode surface (macroelectrode configuration), such that the ions’ diffusion from or to the electrode surface is planar and the CV response yielding current is described as ‘diffusion-limited’ (see Figure 3). However, the diffusion to or from the edge of the macroelectrode is only effective up to a point, therefore the current density and rate of mass transport are larger at the edge and diffusion becomes convergent (equivalent to the microelectrode), which is the case in SECM [44]. Figure 7b provides probe approach curves with working electrode substrate–probe tip distance (*L*). The tip electrode current (*i*_T_) reaches asymptotic behavior with steady-state current following: $i_{T,\infty }=4nFCDa,$ where n is the number of electrons transferred at the electrode tip (O + ne^−^ → R), F is Faraday’s constant, *C* is the concentration or flux of oxidized species, and *D* is the diffusion coefficient limited by the hemispherical region. With the tip approaching the conductive electrode surface, the reduced species formed at the tip is oxidized, resulting in increased tip current following: *i*_T_ > *i*_T,∞_, $i_{t}^{cond}(L)=\frac{i_{T}}{i_{T,\infty }}=[k_{1}+k_{2}/L+k_{3}\exp(k_{4}/L)]$ and creates a regenerative “positive” feedback loop. The opposite effect is observed when scanning an insulating or semiconducting region and diffusion to the electrode is inhibited due to physical obstruction, creating a “negative” feedback loop and decreasing the tip current i.e., *i*_T_ < *i*_T,∞_, $i_{T}^{ins}(L)=\frac{i_{T}}{i_{T,\infty }}=1/[k_{1}+k_{2}/L+k_{3}\exp(k_{4}/L)]$ [46]. Alternatively, the depth profile of the Helmholtz region can be evaluated by changing the polarity and strength of the tip voltage with respect to the substrate [38,41,46].

**Figure 8:**
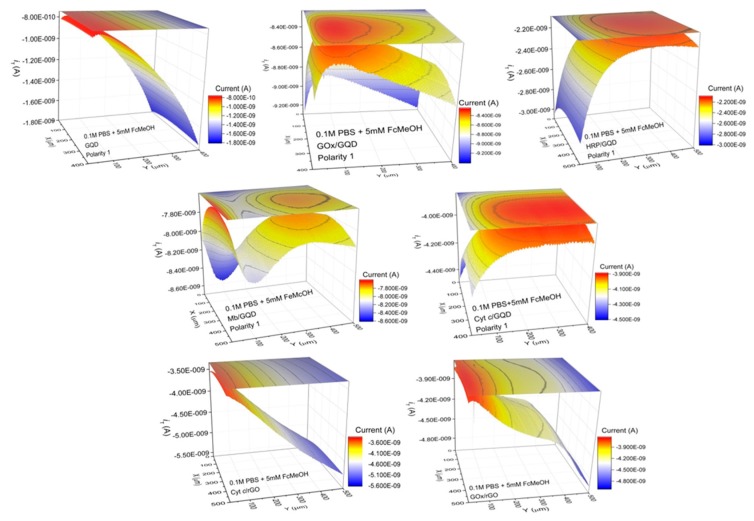
Scanning electrochemical microscopy (SECM) images (500 × 500 μm^2^) of (**a**) GQD; (**b**) GO*_x_*-GQD; (**c**) HRP-GQD; (**d**) Mb-GQD; (**e**) Cyt *c*-GQD; (**f**) Cyt *c*-GO; and (**g**) GO*_x_*-rGO samples displaying probe current distribution in three dimensions with occasional higher/lower current reflecting “highly reactive” electrochemical sites at the solid/liquid interface in redox mediator 5mM FcMeOH (ferrocene methanol) in support electrolyte 0.1 M PBS, conditions: tip voltage *V*_t_ = +0.5 V and substrate voltage *V*_s_ = −0.4 V. A color bar is shown for quantitative values of the current.

The probe approach curves were fitted and plotted in Figure 7b as dashed curves that help to determine the heterogeneous kinetics rate constant at the tip, *k*_ET_ (or k_1_ in the equations above). The *k*_ET_ values of 1.13 × 10^−7^ cm s^−1^, 1.02 × 10^−7^ cm s^−1^, and 0.988 × 10^−7^ cm s^−1^ for GO*_x_*-GQD|GC, GO*_x_*-rGO|GC, and GO*_x_*-GO|GC, respectively, comply with those determined using Laviron’s formalism mentioned above and are within an accuracy of ~0.1%, which is smaller than the typical experimental uncertainty. The tip was polarized at sufficient potential to cause an electrochemical redox reaction (generator) and the current was recorded (collected) over the polarized electrode surfaces at two polarities to visualize electrochemical activity in feedback mode. Figure 8 displays probe tip current distribution as two-dimensional contour ‘heat maps’ and three-dimensional with occasional higher/lower current reflecting “highly reactive” electroactive sites’ distribution, promoted by surface morphology. It is apparent that the samples GO*_x_*-GQD, GO*_x_*-rGO, Cyt *c*-rGO, and Mb-GQD yielded regions of higher electroactive regions with areal site density (~ 60–80 μm^2^), reinforcing the multiple roles played by GQD and rGO in providing edge plane sites than those of GO*_x_*-GO. Thus, changes in the electrochemical activity (heterogeneous electron transfer rate distribution over electrodes’ surfaces) give rise to changes in the feedback (tip) current that make graphene-family nanomaterials usable, once again facilitating their applicability in electrocatalytic glucose sensing applications. Table 1 provides a summary of physicochemical parameters, namely diffusion coefficient (*D*) and electron transfer rate (*k*_ET_) deduced from cyclic voltammograms and SECM, respectively.

## 4. Conclusions

In summary, we have successfully synthesized single-digit graphene quantum dots using solvothermal and hydrothermal techniques, and structurally characterized and explored their traditional and advanced electrochemical properties jointly with an enzymatic glucose biosensor. We identified various dynamic physicochemical processes and determined the associated parameters quantitatively, namely coefficient of diffusion and heterogeneous electron transfer rate. We observed a faster electron transfer with a modified glassy carbon electrode with functionalized GQDs as compared with rGO and GO precursors in the following order: GO*_x_*-GQD|GC >> GO*_x_*-rGO|GC > GO*_x_*-GO|GC. Therefore, GQDs show a great deal of promise, with several key advantages such as: (1) shorter path lengths for electron transportation and shorter diffusion lengths for ion transport permitting feasible electrochemical and electrocatalytic operation; (2) novel and versatile substrates for GO*_x_* immobilization; (3) a high-performance (efficient, highly stable > 95%, a longer shelf life up to months and ultrasensitive amperometric response) enzymatic glucose biosensor, attributed to the larger surface-to-volume ratio, hydrophilic edge sites as well as hydrophobic plane sites as new conduction pathways for ions/electrons, and excellent biocompatibility. We found that GO*_x_* exhibited good peroxidase activity and is recognized as a good electron transporter. SECM provide heterogeneous electron transfer rate and image or map the electrochemical (re)activity site distribution, showing favorable surface ion adsorption with a reasonable site density distribution over GQDs-based electrode surfaces.

## Figures and Tables

**Table 1:** Summary of physicochemical parameters, diffusion coefficient (*D*), and electron transfer rate (*k*_ET_).

Electrode Material	*D* (cm^2^ s^−1^)	*k*_ET_ (cm s^−1^)
GQD (with FcMeOH redox probe)	5.01 × 10^−4^	1.45 × 10^−7^
GO*_x_*-rGO	2.78 × 10^−4^	1.02 × 10^−7^
GO*_x_*-rGO (*I* versus scan rate)	3.03 × 10^−5^	-
GO*_x_*-GO	1.45 × 10^−4^	0.988 × 10^−7^
HRP-GQD	1.19 × 10^−4^	1.15 × 10^−7^
GO*_x_*-GQD	4.43 × 10^−4^	1.13 × 10^−7^

## References

[1] Novoselov K.S., Geim A.K., Morozov S.V., Jiang D., Zhang Y., Dubonos S.V., Grigorieva I.V., Firsov A.A. (2004). Electric field effect in atomically thin carbon films. Science.

[2] Ferrari C., Bonaccorso F., Fal’ko V., Novoselov K.S., Roche S., Bøggild P., Borini S., Koppens F.H., Palermo V., Pugno N. (2015). Science and technology roadmap for graphene, related two-dimensional crystals, and hybrid systems. Nanoscale.

[3] Conway E. (1999). Electrochemical Supercapacitors: Scientific Fundamentals and Technological Applications.

[4] Zhu Y., Murali S., Cai W., Li X., Suk J.W., Potts J.R., Ruoff R.S. (2010). Graphene and graphene oxide: Synthesis, properties, and applications. Adv. Mater..

[5] Chen D., Tang L., Li J. (2010). Graphene-based materials in electrochemistry. Chem. Soc. Rev..

[6] Ping J., Wu J., Wang Y., Ying Y. (2010). Simultaneous determination of ascorbic acid, dopamine and uric acid using high-performance screen-printed graphene electrode. Biosens. Bioelectron..

[7] Kosynkin D.V., Higginbotham A.L., Sinitskii A., Lomeda J.R., Dimiev A., Price B.K., Tour J.M. (2009). Longitudinal unzipping of carbon nanotubes to form graphene nanoribbons. Nature.

[8] Li X.L., Wang X.R., Zhang L., Lee S.W., Dai H.J. (2008). Chemically derived, ultrasmooth graphene nanoribbon semiconductors. Science.

[9] Bagri A., Mattevi C., Acik M., Chabal Y.J., Chowalla M., Shenoy V.B. (2010). Structural evolution during the reduction of chemically derived graphene oxide. Nat. Chem..

[10] Loh K.P., Bao Q., Eda G., Chowalla M. (2010). Graphene oxide as a chemically tunable platform for optical applications. Nat. Chem..

[11] Eda G., Chowalla M. (2010). Chemically Derived Graphene Oxide: Towards Large-Area Thin-Film Electronics and Optoelectronics. Adv. Mater..

[12] Blake P., Brimicombe P.D., Nair R.R., Booth T.J., Jiang D., Schedin F., Ponomarenko L.A., Morozov S.V., Gleeson H.F., Hill E.W. (2008). Graphene-based liquid crystal device. Nano Lett..

[13] Ohno Y., Maehashi K., Yamashiro Y., Matsumoto K. (2009). Electrolyte-Gated Graphene Field-Effect Transistors for Detecting pH and Protein Adsorption. Nano Lett..

[14] Pavlidis V., Patila M., Bornscheuer U.T., Gournis D., Stamatis H. (2014). Graphene-based nanobiocatalytic systems: recent advances and future prospects. Trends Biotechnol..

[15] Dikin D.A., Stankovich S., Zimney E.J., Piner R.D., Dommett G.H.B., Evmenenko G., Nguyen S.T., Ruoff R.S. (2007). Preparation and characterization of graphene oxide paper. Nature.

[16] Baker S.N., Baker G.A. (2010). Luminescent Carbon Nanodots: Emergent Nanolights. Angew. Chem. Int. Ed..

[17] Liu F., Jang M.-H., Ha H.D., Kim J.-H., Cho Y.-H., Seo T.S. (2013). Facile synthetic method for pristine graphene quantum dots and graphene oxide quantum dots: Origin of blue and green luminescence. Adv. Mater..

[18] Lim C.S., Hola K., Ambrosi A., Zboril R., Pumera M. (2015). Graphene and carbon quantum dots electrochemistry. Electrochem Commun..

[19] Martin H.J., Vazquez L., Martinez M.T., Excarpa A. (2014). Controlled chemistry of tailored graphene nanoribbons for electrochemistry: a rational approach to optimizing molecule detection. RSC Adv..

[20] Sekiya R., Uemura Y., Murakami H., Haino T. (2014). White-light-emitting edge-functionalized graphene quantum dots. Angew. Chem. Int. Ed..

[21] Mahasin S.K.A., Ananthanarayanan A., Huang L., Lim K.H., Chen P. (2014). Revealing the tunable photoluminescence properties of graphene quantum dots. J. Mater. Chem. C.

[22] Feng Y., Zhao J., Yan X., Tang F., Xue Q. (2016). Enhancement in the fluorescence of graphene quantum dots by hydrazine hydrate reduction. Carbon.

[23] Suzuki N., Wang Y., Elvati P., Qu Z.-B., Kim K., Jiang S., Baumeister E., Lee J., Yeom B., Bahng J.H. (2016). Chiral Graphene Quantum Dots. ACS Nano.

[24] Hola K., Zhang Y., Wang Y., Giannelis E.P., Zboril R., Rogach A.L. (2014). Carbon dots—Emerging light emitters for bioimaging, cancer therapy and optoelectronics. Nano Today.

[25] Lu J., Yang J., Wang J., Lim A., Wang S., Loh K.P. (2009). One-Pot Synthesis of Fluorescent Carbon Nanoribbons, Nanoparticles, and Graphene by the Exfoliation of Graphite in Ionic Liquids. ACS Nano.

[26] Shen J., Zhu Y., Yang X., Li C. (2012). Graphene quantum dots: Emergent nanolights for bioimaging, sensors, catalysis and photovoltaic devices. Chem. Commun..

[27] Mueller M.L., Yan X., McGuire J.A., Li L.S. (2010). Triplet States and electronic relaxation in photoexcited graphene quantum dots. Nano Lett..

[28] Zhu S., Zhang J., Qiao C., Tang C., Li Y., Yuan W., Li B., Tian L., Liu F., Hu R. (2011). Strongly green-photoluminescent graphene quantum dots for bioimaging applications. Chem. Commun..

[29] Reed M.A. (1993). Quantum Dots. Sci. Am..

[30] Sun H., Wu L., Wei W., Qu X. (2013). Recent advances in graphene quantum dots for sensing. Mater. Today.

[31] Zhang M., Bai L., Shang W., Xie W., Ma H., Fu Y., Fang D., Sun H., Fan L., Han M. (2012). Facile synthesis of water-soluble, highly fluorescent graphene quantum dots as a robust biological label for stem cells. J. Mater. Chem..

[32] Gupta S., Price C., Heintzman E. (2016). Conducting Polymer Nanostructures and Nanocomposites with Carbon Nanotubes: Hierarchical Assembly by Molecular Electrochemistry, Growth Aspects and Property Characterization. J. Nanosci. Nanotechnol..

[33] Gupta S., Heintzman E., Price C. (2016). Electrostatic Layer-By-Layer Self-Assembled Graphene/Multi-Walled Carbon Nanotubes Hybrid Multilayers as Efficient 'All Carbon' Supercapacitors. J. Nanosci. Nanotechnol..

[34] Gupta S., Carrizosa S.B., McDonald B., Jasinski J., Dimakis N. (2017). Graphene-family nanomaterials assembled with cobalt oxides and cobalt nanoparticles as hybrid supercapacitive electrodes and enzymeless glucose detection platforms. J. Mater. Res..

[35] Gupta S., Aberg B., Carrizosa S.B., Dimakis N. (2016). Vanadium pentoxide nanobelt-reduced graphene oxide nanosheet as high-performance pseudocapacitive electrodes: AC impedance spectroscopy data modeling and theoretical calculations. Materials.

[36] Gupta S., VanMeveren M., Jasinski J. (2015). Investigating Electrochemical Properties and Interfacial Processes of Manganese Oxides/Graphene Hybrids as High-Performance Supercapacitor Electrodes. Int. J. Electrochem. Sci..

[37] Gupta S., Wood R. (2017). Development of FRET biosensor based on aptamer/functionalized graphene for ultrasensitive detection of bisphenol A and discrimination from analogs. Nano-Struct. Nano-Objects.

[38] Gupta S., Irihamye A. (2015). Probing the nature of electron transfer in metalloproteins on graphene-family materials as nanobiocatalytic scaffold using electrochemistry. AIP Adv..

[39] Wu P., Shao Q., Hu Y., Jin J., Yin Y., Zhang H., Cai C. (2010). Direct electrochemistry of glucose oxidase assembled on graphene and application to glucose detection. Electrochim. Acta.

[40] Zeng G., Xing Y., Gao J., Wang Z., Zhang X. (2010). Unconventional Layer-by-Layer Assembly of Graphene Multilayer Films for Enzyme-Based Glucose and Maltose Biosensing. Langmuir.

[41] Park S., An J., Potts R.J., Velamakanni A., Murali S., Ruoff R.S. (2011). Hydrazine-reduction of graphite- and graphene oxide. Carbon.

[42] Sheng Z., Song L., Zheng J., Hu D., He M., Zheng M., Gao G., Gong P., Zhang P., Ma Y. (2013). Protein-assisted fabrication of nano-reduced graphene oxide for combined in vivo photoacoustic imaging and photothermal therapy. Biomaterials.

[43] Mosa I.M., Pattammattel A., Kadimisetty K., Pande P., El-Kady M.F., Bishop G.W., Novak M., Kaner R.B., Basu A.K., Kumar C.V. (2017). Ultrathin Graphene–Protein Supercapacitors for Miniaturized Bioelectronics. Adv. Energy Mater..

[44] Pan D., Zhang J., Li Z., Wu M. (2010). Hydrothermal route for cutting graphene sheets into blue-luminescent graphene quantum dots. Adv. Mater..

[45] Bard J., Mirkin M.V. (2001). Scanning Electrochemical Microscopy.

[46] Wang S., Cole I.S., Zhao D., Li Q. (2016). The dual roles of functional groups in the photoluminescence of graphene quantum dots. Nanoscale.

[47] Efros L., Rosen M. (2000). The Electronic Structure of Semiconductor Nanocrystals. Annu. Rev. Mater. Sci..

[48] Gupta S., Saxena A. (2009). Nanocarbon materials: Probing the curvature and topology effects using phonon spectra. J. Raman Spectrosc..

[49] Dresselhaus M.S., Eklund P.C. (2000). Phonons in carbon nanotubes. Adv. Phys..

[50] Wu B., Hou S., Miao Z., Zhang C., Ji Y. (2015). Layer-by-layer self-assembling gold nanorods and glucose oxidase onto carbon nanotubes functionalized sol-gel matrix for an amperometric glucose biosensor. Nanomaterials.

[51] Laviron E. (1979). General expression of the linear potential sweep voltammogram in the case of diffusionless electrochemical systems. J. Electroanal. Chem..

[52] McCreery R.L. (2008). Advanced Carbon Electrode Materials for Molecular Electrochemistry. Chem. Rev..

[53] Shangguan X., Zhang H., Zheng J. (2008). Direct electrochemistry of glucose oxidase based on its direct immobilization on carbon ionic liquid electrode and glucose sensing. Electrochem. Commun..

[54] Razmi H., Rezaei R.M. (2013). Graphene quantum dots as a new substrate for immobilization and direct electrochemistry of glucose oxidase: Application to sensitive glucose determination. Biosens. Bioelectron..

[55] Huang Y., Zhang W., Xiao H., Li G. (2005). An electrochemical investigation of glucose oxidase at a CdS nanoparticles modified electrode. Biosens. Bioelectron..

[56] Guo S., Zhang S., Wu L., Sun S. (2012). Co/CoO nanoparticles assembled on graphene for electrochemical reduction of oxygen. Angew. Chem. Int. Ed..

[57] Zhang Y., Wu C., Zhou X., Wu X., Yang Y., Wu H., Guo S., Zhang J. (2013). Graphene quantum dots/gold electrode and its application in living cell H_2_O_2_ detection. Nanoscale.

[58] Gupta S., Banaszak A., Smith T., Dimakis N. (2017). Molecular sensitivity of metal nanoparticles decorated graphene-family nanomaterials as surface-enhanced Raman scattering (SERS) platforms.

[59] Li J., Tan S.N., Ge H. (1996). Silica sol-gel immobilized amperometric biosensor for hydrogen peroxide. Anal. Chim. Acta.

[60] Gupta S., Carrizosa S.B. (2016). Insights into electrode/electrolyte interfacial processes and the effect of nanostructured cobalt oxides loading on graphene-based hybrids by scanning electrochemical microscopy. Appl. Phys. Lett..

[61] McGovern W.R., Anariba F., McCreery R.L. (2005). Importance of oxides in carbon/molecule/metal molecular junctions with titanium and copper top contacts. J. Electrochem. Soc..

[62] Brownson D.A.C., Kampouris D.K., Banks C.E. (2012). Graphene electrochemistry: Fundamental concepts through to prominent applications. Chem. Soc. Rev..

